# Factors Associated With Implementation of Biomarker Testing and Strategies to Improve Its Clinical Uptake in Cancer Care: Systematic Review Using Theoretical Domains Framework

**DOI:** 10.1200/PO-25-00063

**Published:** 2025-10-09

**Authors:** Rehana A. Salam, Kate L.A. Dunlop, Tuba N. Gide, James Wilmott, Andrea Smith, Anne E. Cust

**Affiliations:** ^1^ The Daffodil Centre, The University of Sydney, A Joint Venture with Cancer Council NSW, Sydney, NSW, Australia; ^2^ Melanoma Institute Australia, The University of Sydney, Sydney, NSW, Australia; ^3^ Charles Perkins Centre, The University of Sydney, Sydney, NSW, Australia; ^4^ Faculty of Medicine and Health, The University of Sydney, Sydney, NSW, Australia

## Abstract

**PURPOSE:**

We aimed to synthesize evidence on factors associated with implementation of biomarker testing and strategies to improve its clinical uptake in cancer care.

**METHODS:**

MEDLINE, EMBASE, and CENTRAL databases were searched until July 7, 2024. We used the Theoretical Domains Framework to report factors associated with implementation of biomarker testing from clinicians' and patients' perspectives and identify strategies to improve its clinical uptake.

**RESULTS:**

We included 77 studies: 47 studies reported factors from clinicians' perspective, 33 studies reported patients' perspective, 23 studies reported implementation strategies, and seven studies evaluated effectiveness of interventions to facilitate implementation and improve uptake. Clinicians reported inconsistent knowledge and skills related to interpreting results of biomarker testing, making treatment recommendations, and communicating findings of uncertainty and its implications for patients. Patients reported gaps in their knowledge about biomarker testing, how it related to treatment, and the research processes. Long turnaround times, lack of coverage by health insurance plans, and logistical constraints also impaired implementation. Concerns associated with inappropriate use of biomarker testing in unvalidated populations, safety and efficacy profiles of the corresponding immuno-oncology agents, lack of access to corresponding trials, and setting potentially unrealistic expectations for patients regarding their prognosis were highlighted. There are scarce data on strategies and interventions to facilitate implementation and improve uptake of biomarker testing. Setting up an institutional tumor board, multidisciplinary team coordination, and formal ongoing education were the most frequently reported strategies to facilitate implementation. Educational interventions were reported to be feasible, acceptable, and increased knowledge.

**CONCLUSION:**

This review highlights many factors that are amenable to aid implementation and clinical uptake. However, there is a need to evaluate strategies addressing uncertainties and barriers.

## INTRODUCTION

Precision care models have revolutionized cancer treatment by offering patients an opportunity to receive personalized treatment, thus improving tumor response to treatment and survival.^1^ However, equitable and timely access to optimal therapy based on biomarker results remain challenging because of a myriad of reasons ranging from the lack of validated biomarkers to standardized integration of biomarker testing into the existing clinical practices.^2-5^ Moreover, the paradigm shift to biomarker-driven treatment has led to an increased number of basket trials, umbrella trials, and platform trials to increase pace and scale of clinical development, simultaneously raising some procedural concerns.^6,7^ Despite the progress in biomarker-informed treatment, therapeutic benefit is not always guaranteed, which further challenges its implementation.

Notwithstanding the complexities associated with biomarker development and validation, its implementation into routine clinical practice also remains challenging. Clinicians and patients face distinct concerns related to the role and value of biomarker testing in informing personalized prognosis and treatment.^8^ Although patients and physicians share similar priorities regarding clinical outcomes, there are often differences in their expectations and the potential results from biomarker-driven treatment may not align with patient-anticipated outcomes.^9^ For example, patients might have disproportionate expectations of extending life, reducing symptoms, avoiding toxicities associated with therapy or cure and might not be cognizant of the fact that personalized actionable targets are still in the exploratory phase of clinical research.^9^ Both patients and health care providers face uncertainty regarding the choice of biomarker testing approaches, optimal timing to conduct biomarker testing, and available treatment options. Furthermore, biomarker testing may potentially help to identify patients who will benefit the most from a treatment; however, it does not guarantee treatment benefit nor necessarily mean that the patients without the biomarker will not receive treatment benefits. Shared decision making to decide the best course of action based on benefits, harms, and preferences also remains an evolving field. The associated nuances of the biomarker testing along with the patients' health condition tend to alter patients' understanding and recall related to the knowledge and the results despite their motivation to understand.^10^

Expanded biomarker testing has the potential to become a part of modern clinical practice; however, clinical uptake remains low^11-14^ owing to low awareness,^15^ lack of evidence-based practice guidelines, fragmented insurance coverage, and inadequate reimbursement.^16,17^ With the increasing emphasis on personalized cancer treatment, it is important to understand factors that might influence clinical uptake of biomarker testing from the clinicians' and patients' perspective. Without an in-depth understanding of these factors, highlighting the clinical utility of biomarker testing and its limitations, the implementation of a precision care model based on biomarker testing may fail to deliver expected benefits. Therefore, systematically identifying these factors from both health professionals' and patients' perspectives is important and timely. Our aim was to synthesize existing evidence on the factors associated with the implementation of biomarker testing in cancer care from clinicians' and patients' perspectives. We also aimed to identify existing evidence on strategies and interventions to facilitate the implementation and improve clinical uptake of biomarker testing in cancer care. We used the Theoretical Domains Framework (TDF) to identify and better understand the associated factors and how they influence both clinicians' and patients' behaviors to inform implementation strategies that can be used to facilitate implementation.^18^ Evidence generated from this review has the potential to assist in designing targeted strategies and interventions to reduce implementation barriers and improve clinical uptake of biomarker testing.

## METHODS

### Criteria for Considering Studies for This Review

We followed the Preferred Reporting Items for Systematic Reviews and Meta-Analyses guidelines.^19^ Table 1 summarizes the eligibility criteria.

**TABLE 1.** Eligibility Criteria

Eligibility Criteria	Inclusion Criteria	Exclusion Criteria
Study design	Quantitative (interventional or noninterventional), qualitative, or mixed methods studies	Editorials, opinions, systematic/narrative reviews, modeling studies, guidelines, case studies, case reports, commentaries, letters, conference abstracts, books, gray literature, and consensus studies
Study context	Studies conducted in any country targeting any type and stage of cancer	
Population/participants	Clinicians involved in cancer care and/or patients with cancer of any age and sex	Studies focusing on screening healthy population for inherited mutations
Concepts	Factors associated with the implementation and uptake of biomarker testing in cancer care from clinicians' and patients' perspectives Strategies or interventions to facilitate implementation and improve clinical uptake of biomarker testing in cancer care	Clinical trials on biomarker identification or validation Studies reporting the impact of molecular tumor boards on disease-specific outcomes (eg, frequency of actionable molecular alterations identified) Descriptive studies reporting the process of implementing biomarker testing platforms, describing webservers, or data sharing platforms without reporting any outcomes
Publication date	Published until July 7, 2024	
Language	English	

### Types of Studies

We included quantitative, qualitative, or mixed methods studies assessing the factors associated with the implementation of biomarker testing in cancer care from clinicians' and patients' perspectives. We also included studies evaluating strategies or interventions to facilitate implementation and improve clinical uptake of biomarker testing in cancer care. Since variable terms are currently being used for biomarker testing in the literature, for this review, biomarker testing included tumor testing, tumor genetic testing, genomic testing or genomic profiling, molecular testing or molecular profiling, somatic testing, and/or tumor subtyping. We excluded the following studies from our review:

1. Clinical trials on biomarker identification or validation since the review aimed to synthesize evidence on factors associated with its implementation and clinical uptake;
2. Studies focusing on genetic testing for inherited mutations in healthy populations since the target population for this review was patients with cancer;
3. Studies reporting the impact of molecular tumor boards on only disease-specific outcomes (eg, frequency of actionable molecular alterations identified) without reporting any factors associated with implementation or clinical uptake since the aim of this review was to identify strategies or interventions to facilitate implementation of biomarker testing and improve clinical uptake;
4. Descriptive studies reporting the process of implementing biomarker testing platforms and describing webservers or data sharing platforms without reporting any associated factors;
5. Consensus studies using methodologies such as Delphi and nominal group technique.

### Types of Participants

We included studies conducted among clinicians involved in cancer care and/or patients diagnosed with cancer, of any age and sex. Studies targeting any type and stage of cancer were eligible for inclusion.

### Data Collection and Analysis

#### Selection of Studies

We conducted an initial search on MEDLINE, EMBASE, and CENTRAL databases in November 2023, and an updated search was conducted on July 7, 2024. The search strategy is provided in Appendix Table A1. Search results were exported into EndNote, deduplicated, and uploaded into Covidence (an online web-based systematic review tool) for screening.^20^ One review author (R.A.S.) screened the titles and abstracts for inclusion. Two review authors (R.A.S. and K.L.A.D.) independently screened the full texts for inclusion. Any discrepancies during full-text screening were resolved by consensus. Reasons for exclusion at the full-text screening stage were recorded. We manually checked reference lists of primary studies and review articles for any additional references that were not captured in the database search.

#### Data Extraction

Data were extracted for key variables including study characteristics and outcomes in a standardized data collection form. We extracted data on the following study characteristics:

1. Study details: author, title, journal, publication year.
2. Study methods: study design, duration of study, study location, sample size, sampling procedure, study settings, method of data collection, and analysis.
3. Participants: types of participants (clinicians, patients), inclusion criteria, exclusion criteria.
4. Biomarkers: type of cancer, cancer stage, type of biomarker testing.
5. Factors: factors associated with implementation of biomarker testing from clinicians' and patients' perspective.
6. Strategies or interventions: description of any strategy or intervention to facilitate implementation and improve clinical uptake of biomarker testing and reported outcomes including any measure of effectiveness as defined by the study author.
7. Additional information: funding sources, study limitations, and notable conflicts of interest.

#### Data Synthesis

We used the TDF to extract and classify factors associated with implementation into the 14 domains. The 14 domains of TDF along with their respective constructs are detailed in Appendix Table A2. In assigning the data to the most relevant TDF domain, relevant contextual information reported was cross-referenced to TDF constructs to check for alignment. R.A.S. classified and extracted the data into the domains, whereas K.L.A.D. and A.S. ensured consistent interpretation of the domains and the data extracted. Strategies and interventions, where reported, were also extracted and classified according to the TDF domains.

#### Quality Assessment of Included Studies

We assessed the risk of bias in the included studies using the National Institute of Health NHLBI tool^21^ for cross-sectional and before/after studies, the Joanna Briggs checklist for qualitative research appraisal tool^22^ for qualitative studies, the mixed methods appraisal tool^23^ for the mixed methods studies, and the Cochrane risks of bias tool^24^ for randomized studies. Each of the included studies were assessed as per the specific criteria for the respective study design and were classified as having overall low risk, some concerns, or high risk.

## RESULTS

**FIG 1.**
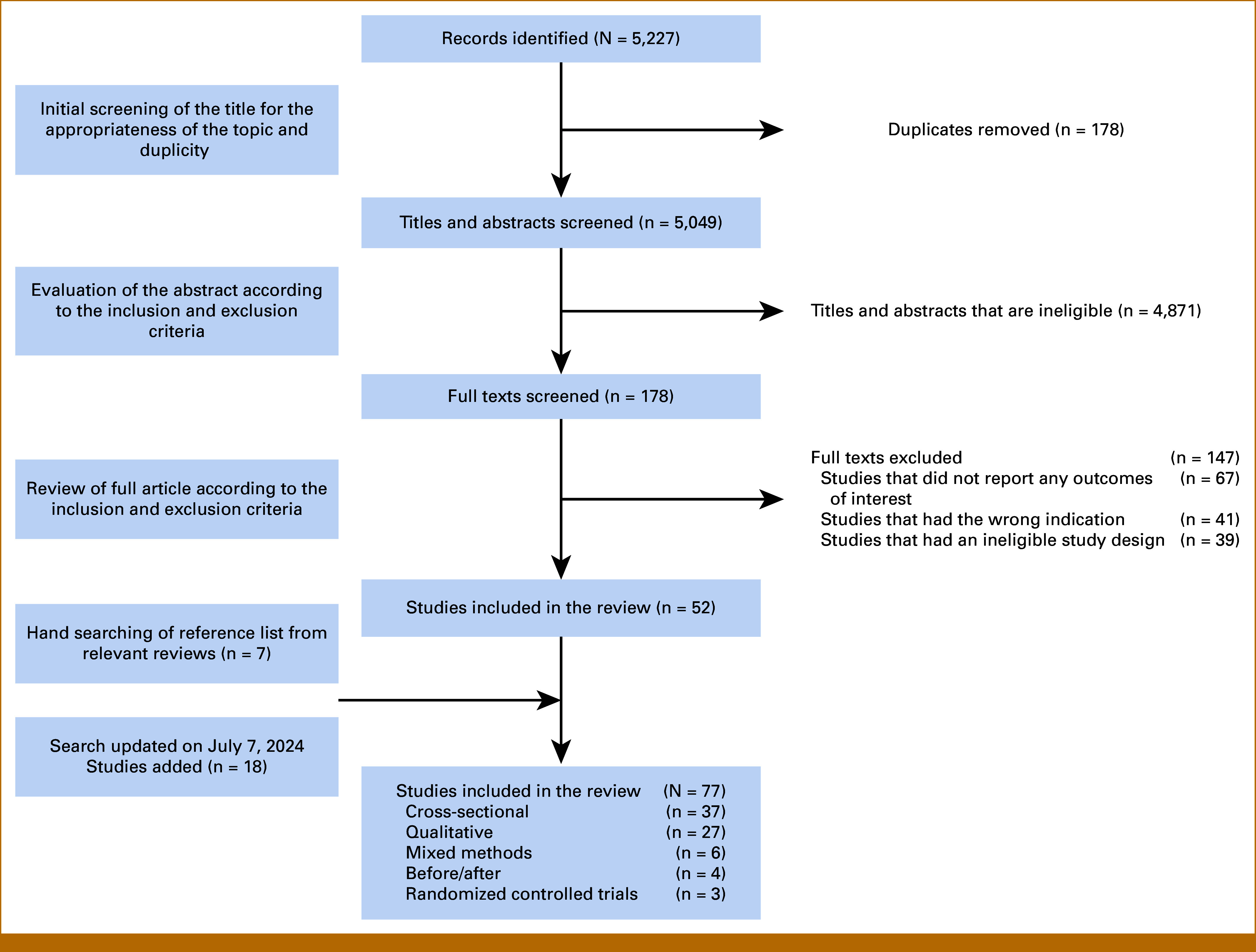
Search flow diagram.

Our search yielded a total of 5,227 records, and we screened 5,049 titles and abstracts after removing duplicates. Excluding ineligible records based on title and abstract screening, we screened 178 full texts. We included 77 studies in our review, of which 37 studies were cross-sectional, 27 studies were qualitative studies, six studies were mixed methods studies, four studies were before/after studies, and three studies were randomized controlled trials. Figure 1 depicts the search flow diagram.

### Characteristics of Included Studies

The included studies were conducted between 2009 and 2024. Over half of the studies (56%) were conducted in the United States, followed by Canada (12%) and Australia (5%). Over 95% of the included studies were conducted in high-income countries, whereas 5% of the studies were conducted in low- and middle-income countries including Lebanon, India, Pakistan, and Rwanda. Participants in the majority of the included studies were clinicians involved in cancer care; however, a few studies included other health care professionals such as nurses and laboratory technicians. Table 2 summarizes the characteristics of the included studies.

**TABLE 2.** Characteristics of Included Studies

Study Characteristic	No. (%)
Total	77 (100)
Study country	
United States	43 (55.8)
Canada	09 (11.7)
Australia	04 (5.2)
Japan	03 (3.9)
The Netherlands	02 (2.6)
Singapore	02 (2.6)
Germany	01 (1.3)
France	01 (1.3)
United Kingdom	01 (1.3)
Italy	01 (1.3)
Belgium	01 (1.3)
Ireland	01 (1.3)
Lebanon	01 (1.3)
India	01 (1.3)
Pakistan	01 (1.3)
Rwanda	01 (1.3)
Multicountry	04 (5.2)
Study design	
Cross-sectional	37 (48.0)
Qualitative	27 (35.0)
Mixed	06 (7.8)
Before/after	04 (5.2)
Randomized controlled trials	03 (3.9)
Cancer type	
Multiple	48 (62.3)
Breast	15 (19.5)
Lung	07 (9.1)
Head and neck	03 (3.9)
Pediatric hematology-oncology	02 (2.6)
Lymphoid	01 (1.3)
GI	01 (1.3)
Brain	01 (1.3)
Colorectal cancer	01 (1.3)
Pediatric cancer	01 (1.3)

### Quality of Included Studies and Outcomes Reported

**FIG 2.**
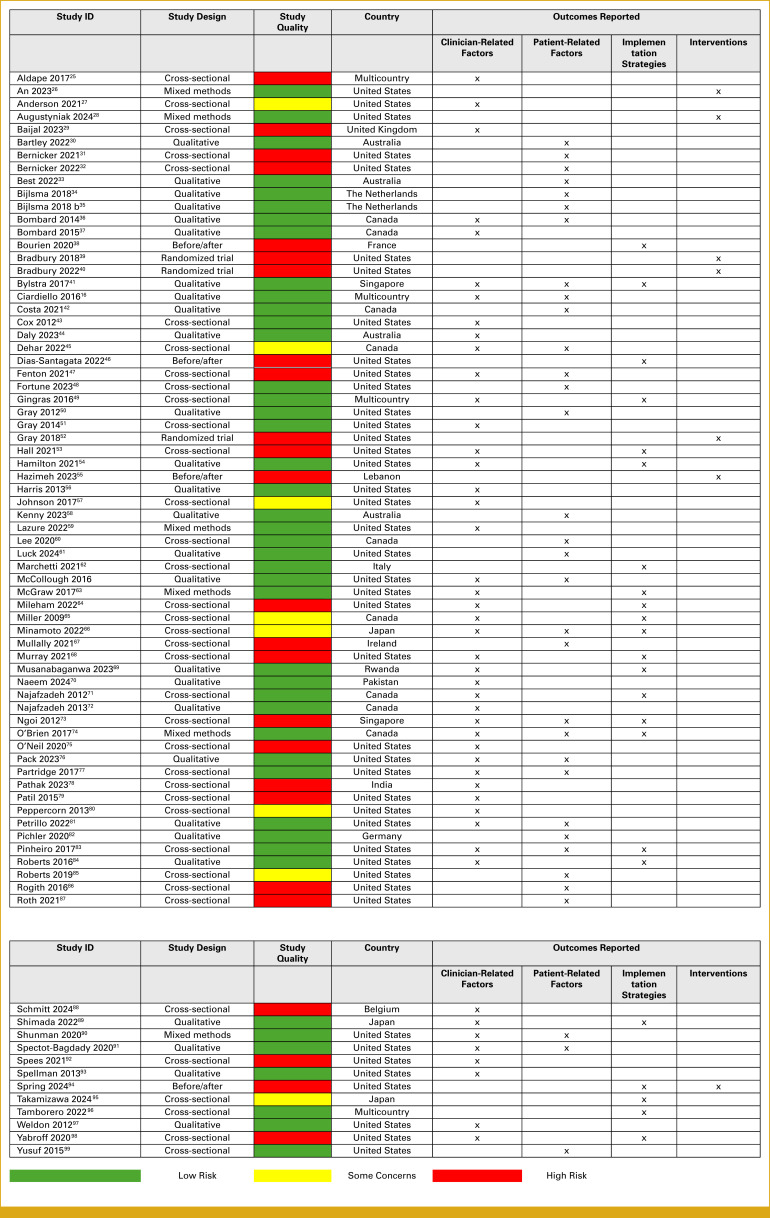
Quality assessment of the included studies and outcomes reported.

Figure 2 summarizes the quality of included studies and the outcomes reported.^16,25-50,51-75,76-99^ Forty-seven studies reported factors from clinicians' perspective, 33 studies reported factors from patients' perspective, 23 studies reported strategies for implementation, whereas seven studies evaluated effectiveness of interventions to facilitate implementation of biomarker testing. The majority of cross-sectional studies were found to be at high risk of bias or had some concerns because of low participation rates, small sample size, study power, and bias because of confounding. Qualitative and mixed methods studies were found to be of good quality. Before/after studies were found to be of poor quality because of small sample size, lack of blinding, inappropriate use of statistical methods, and outcome measurement at a single point in time. Randomized controlled trials were found to be at high risk of bias because of bias arising from the random assignment process and missing outcome data.

### Findings

We report findings from the included studies categorized across all 14 domains of the TDF for factors associated with implementation of biomarker testing from clinicians' and patients' perspectives, strategies, and interventions to facilitate implementation and improve uptake of biomarker testing. These findings are also summarized in Table 3.

**TABLE 3.** Clinician-Related and Patient-Related Factors, Strategies, and Interventions According to the Domains of the Theoretical Domains Framework

Domains^18^	Clinician-Related Factors	Patient-Related Factors	Implementation Strategies	Interventions
Knowledge (an awareness of the existence of something)	Clinicians specializing in the field were aware of the test^69,80^; however, limited and inconsistent knowledge was reported among other health professionals^41,66,70^ Knowledge around how to order the test, interpreting test results, safety profiles of immuno-oncology agents, and information sources to guide patients were reported to be limited even among oncologists^53,59,65^ Existing knowledge was based primarily on informal education^53,59,65^ Clinicians reported uncertainties and had low confidence around their patients' knowledge and their patients' ability to comprehend the test and its implications^16,27,37^ Knowledge was reported to be limited consistently in academic and community practices^64^	Patients reported deficits in their own knowledge around testing, what the test meant, and how it related to their treatment, and the research processes^30,60,67,73,86,87^ Patients highlighted the need for information^33,41,48^ Patients with greater health literacy and income reported high knowledge^48,86^ Patients stated that they wanted their doctors to explain it to them^16^	Additional training including formal and ongoing education for clinicians^41,53,64,65,74,89^ Improved genetic literacy among patients^17^ Online resources were suggested as a practical alternative to formal training sessions^71^	Digital educational intervention improved patients' and clinicians' knowledge about precision oncology and clinical trials^26,40,55^ An online accredited education program for health care providers led to improved knowledge about identification of mutations and selection of appropriate treatment^28^
Skills (an ability or proficiency acquired through practice)	Clinicians reported limited skills in assessing patient knowledge and information preferences, counseling and educating patients about implications, setting patient expectations, and communicating rationale for treatment selection^41,49,54,59,89^ Limited skills to interpret the results and make treatment recommendations^49,51,53,59,72^ Low skills leading to inadequate tissue^59,64^ Clinicians reported lack of patient education materials, preferably print-based rather than a web tool, to help communicate treatment recommendations to share with patients/families^75,92^ Factors associated with improved skills were high patient volume,^49,75^ professional experiences,^63^ ease of use,^79^ and improved confidence in interpreting test results^57^ Skills were reported to be higher among academic *v* community providers^64^	Patients felt incompetent explaining the test to their relatives and highlighted the importance of understanding their results to communicate with relatives and reduce their uncertainties^30,33,41^	Educating physicians to provide better explanations to patients and communicating uncertainties^54,71,89^ Having institutional guidelines^49^ International academic exchange programs to train health professionals^69^ Trainings for laboratory technicians^69^ Electronic medical record alerts for genomic tests^98^	Interactive genomic reports may improve physicians' ability to accurately assess genomic data and increase report-related satisfaction^52^
Social/professional role and identity (a coherent set of behaviors and displayed personal qualities of an individual in a social or work setting)	Clinicians reported lack of expert consensus, professional recommendations, clinical guidelines, and protocols on the use of genomic tests^71-73,80^ Oncologists are expected to be the gatekeepers for providing (or not providing) information regarding testing, interpretation of results, and all the available treatment options^16,36,74^ There were uncertainties regarding whether medical oncologists or surgeons should order testing^93^ Clinicians stated that the field is evolving, and further evidence is required to understand the test and the findings which was beyond their role as oncologists^41^ General physicians reported having a trusting relationship with patients and that they might want to discuss their testing results, treatment options, and decision making with their physicians^74^ Clinicians believed that the treatment decision should involve the doctor, the multidisciplinary team, and the patient^16^ and expected clinical genetics professionals to have better knowledge and participate more actively in decision making^89^ Insufficient interdepartmental cooperation, especially between oncology and clinical genetics departments^66^ Varying definitions of precision oncology depending on the subspeciality^54^ Recent training, being a researcher, peer use, and availability of molecular pathologists were associated with higher levels of awareness and testing^64,68,73,84^	Patients valued expertise and perceived researchers as holding the greatest knowledge, expressing reliance and trust in the clinical enterprise of their health care provider^30,90^ Unsupportive patient-family communication undermined patient's interest in taking the test^35,61^	Setting up an institutional tumor board and multidisciplinary team coordination in treatment decision making with clear lines of communications and better collaboration^49,64,74,84,89,96^ More time allocated for clinicians to conduct research and keep up-to-date with the recent research^49^ Training of other health professionals including nurses and primary caregivers^69^ Adoption of an expert-agreed process to systematically link test results with clinical actions, structured data capture, and use of interactive patient reports^96^	Implementing a real-time informal consultation and prescreening assessment for patients treated by community, and academic medical oncologists by a virtual molecular and precision medicine clinic enhanced efficiency in referrals, improved patient satisfaction, interpretation of genomic testing, and clinical trial enrollment for patients^94^
Beliefs about capabilities (acceptance of the truth, reality, or validity about an ability, talent, or facility that a person can put to constructive use)	Clinicians reported variable confidence in their ability to effectively integrate testing into clinical management^25,27,51,53^ Clinicians reported low self-efficacy to decide whether testing was indicated, interpreting and communicating test results to the patients, explaining findings of uncertainty, and making treatment recommendations based on results^43,51,53,57,65,74,92^ The decision for testing was influenced by clinicians' experiences, attitudes, perceived barriers, and confidence in using test results^51,75,84^ Clinicians reported confusion regarding the consent process^91^ and highlighted the need to separate clinical and research consent^90^	Higher self‐reported level of understanding was associated with higher odds of endorsing direct benefits^85^ Patients were confused regarding the informed consent^91^	Further training on identifying indications for ordering the test, interpreting and individualizing clinical management based on results, and counseling on the risks and benefits of testing^68^ Educating about ethical and financial considerations surrounding testing and communicating test results^68^	Digital educational intervention improved patients' and clinicians' scores for decision self-efficacy for testing and targeted therapy^26^ An online accredited education program for health care providers led to improved confidence in identification of mutations, selection of appropriate treatment, and ability to engage patients in shared decision making and management of adverse events^28^
Optimism (the confidence that things will happen for the best or that desired goals will be attained)	Clinicians reported that testing could potentially lead to a range of possible treatment options and potentially better outcomes for patients and would be useful in their practice^54,72,74,93^ Clinicians were mostly optimistic and willing to integrate testing into their practice, provided that they have access to appropriate training and resources^69,70,72^ Some concerns were raised around inappropriate use of the test in unvalidated groups of patients^37^ and low likelihood of identifying clinically actionable results^27^ Oncologists' initial framing sometimes sets high expectations for the benefits of targeted therapy for the patients, and hence, aligning patients' expectations with the realities was considered important^54,81^ Clinicians underestimated that their patients would be willing to delay the treatment for test results^16^ In low-resource settings, physicians were not very hopeful for implementation and availability for all patients in near future^70^	A majority of patients expressed interest in taking the test and were optimistic about contributing to advancing research to generate more reliable information that could be used to inform prognosis and improve survival even if efforts were unlikely to provide them with personal benefit^30,33,42,67,73,85,90^ Patients reported high expectations of the test results and considered them a backup when other treatment approaches failed, and sometimes oncologists' framing of detection of a targetable mutation set these high expectations^33,81,87^ Patients reported willingness to delay treatment to allow for receiving test results^16^ Patients reported concerns around receiving test results of uncertain significance^30^ and expressed their willingness to have more formal counseling before pursuing the test^45^		
Beliefs about consequences (acceptance of the truth, reality, or validity about outcomes of a behavior in a given situation)	Clinicians felt that the testing was beneficial and worthwhile for the management of patients^25,27,37,57,65,73^ and their intention to use the test increased with their perceptions about its usefulness^79^ Despite valuing the test, oncologists did not report that it fundamentally altered their practice and viewed it as a tool for evidence-based practice that was still being developed^37,74,75,81,100^ Clinicians reported concerns around over-reliance on the test,^37^ testing inappropriately,^93^ uncertainty in validity,^54,72^ lack of evidence of benefit,^49,63,73,80^ or lack of evidence comparing the safety and efficacy of agents.^59,95^ Clinicians had reservations about testing either when precision treatments are not available or they do not have access to corresponding clinical trials, and hence, testing did not affect patients' treatment options^49,54,73,78,89^ Clinicians also raised concerns around patients misperceptions of likelihood of a cure or treatment side effects^54^	Patients reported that they considered the test useful despite the associated uncertainties^33,42,60^ Patients reported concerns regarding psychological harm^50^	Development of a framework to guide decisions about uncertain results^63^	
Reinforcement (increasing the probability of a response by arranging a dependent relationship, or contingency, between the response and a given stimulus)	Clinicians were either unaware if there were policies in place or reported lack of clear guidelines regarding clinical indications and implementation^65,69^ Some reported lack of a uniform reporting system^78^	Patients reported the need for more strategies to promote understanding of the test results^33^	Having institutional guidelines^49^	
Intentions (a conscious decision to perform a behavior or a resolve to act in a certain way)	Clinicians were intent on testing on the basis of clinical utility, that is, if it was likely to add value to the clinical decision-making process or was part of treatment guidelines^16,37,45,47,71,83^ Clinicians intended to become competent in ordering and interpreting test^68^ Intentions were affected by relatively low priority and uncertainty around secondary findings of hereditary (germline) mutations^69,89^	The potential to guide treatment and contribute to research influenced decisions to undergo testing^41,45,83,85,99,100^ Patients reported varying preferences for return of information based on test results and preferred selective subsets of information that could be acted upon^34,42^		An online accredited education program for health care providers led to improved intentions of physicians to change in practice regarding identification of mutations, selection of appropriate treatment, and ability to engage patients in shared decision making and management of adverse events^28^
Goals (mental representations of outcomes or end states that an individual wants to achieve)	Clinicians stated that future research would facilitate better understanding by examining patients' comprehension and how to best communicate testing and results^73,84^	Patients preferred the most effective course and discounted toxicity and valued increased benefit^77^	Future research should include feasibility studies to identify the required technologies and human resources to be trained to drive the implementation^69^	
Memory, attention, and decision processes (the ability to retain information, focus selectively on aspects of the environment, and choose between two or more alternatives)	Clinicians reported that their patient's poor health led to a compromised understanding when burdened with stressful information and details of the test results could lead to information overload for the patients^59,81,84,89^	Patients preferred straightforward communication, education, and counseling to make informed decisions with written information and time to review this information before decision making^34,42,45,47,83^ Patients stated that the results are cognitively complex to understand and make decisions and hence could create unnecessary confusion and worry without receiving relevant information^35,41^ Patients preferred shared clinical decision making and saw their oncologist as a key partner in their decision-making process^33,42^ Patients believed that they needed more control in their lives and agreed that test results offered them more control^35,67^ Patients required psychological support and the presence of family^34^	Easy-to-understand patient education resources including pamphlets, websites, and scripts, to communicate information related to the test and test results^59,83^	
Environmental context and resources (any circumstance of a person's situation or environment that discourages or encourages the development of skills and abilities, independence, social competence, and adaptive behavior)	Clinicians were concerned with the logistics of implementation including excessive turnaround times for test results,^16,29,32,64,97^ administrative requirements,^27,36^ lack of adequate staff,^66,84^ and high costs^16,37,70,73,88^ Lack of insurance coverage^16,31,54,65,66,69,79,84,97^ Clinicians also expressed that they had limited time in clinics^41,54,59,92^ Organizational factors including lack of funding,^49,65^ infrastructure and technologies^69,84^ Concern for impact on privacy or confidentiality^57,72,91^ Follow-up and consequent management of incidental/secondary findings^54^ Lack of ethical, legal, and social frameworks^69^	Patients were willing to undergo the test given that it was covered by insurance; however, they stated privacy concerns^30,34,41,50,73,91,99^ Patients reported delays, confusion, and increased anxiety associated with the administrative requirements^36^	Increased resources and training for clinical genetic professionals^41,89^ Advocacy and mobilization of funds and institutionalizing legal frameworks (protocols, policy and procedure manuals, and guidelines)^69,89^ Update health sector policies and setting up interinstitutional exchange programs to facilitate implementation^69^ Automating the interpretation and reporting of sequencing results decrease the need for time-consuming manual procedures that are prone to errors^96^	
Social influences (those interpersonal processes that can cause individuals to change their thoughts, feelings, or behaviors)	Clinicians reported that mass media increased awareness; however, it led ineligible patients to feel that they were unfairly being denied access to an important test^36,54,81^ and was sometimes prone to aggressive marketing^37,72^ Clinicians also highlighted some equity issues like access in rural areas^54,72^ Discrimination based on the genomic information (by insurance companies, the health care system, and employers)^72,73^ Patient-reported and physician-assessed sources of information about diagnosis and treatment differed considerably^16^	Patients reported that having a positive and trusting relationship with their health care providers reduced uncertainties while insufficient or quick information or complex language to communicate results led to increased uncertainty about results and treatment recommendations^16,30,33,41,42,61^ Sources of information about the test differed; however, mass media was reported as a catalyst for access to information^16,36,81^ Patients reported that the communication around testing was limited and cursory^33,41^ Patients felt that test results could lead to discrimination and hence suggested limited access to this information for employers and insurance companies, whereas they were happy to share information with family members^50,67^ The way patients learned about the test (early *v* late) led to perceived inequities in access which negatively affected the doctor–patient relationship^36^		
Emotion (a complex reaction pattern, involving experiential, behavioral, and physiological elements, by which the individual attempts to deal with a personally significant matter or event)	Psychological impact and need for psychological support for patients^57,66,89,100^ Clinicians stated that high expectations from patients led to patient disappointment and anger in the case of nonactionable findings^54,81^ Oncologists anchored on stories of longer-term survivors and avoided prognostic conversations to help patients to cope with their cancer^81^ Navigating clinical trials is burdensome for the clinicians^54^	Patients reported emotional burden associated with nonactionable or unsolicited findings and their prognosis in the absence of targetable treatment options^34,35,42,50^ Patients also reported that knowing the test results was more important than not knowing regardless of the potential anxiety and relieved treatment uncertainty to some degree^30,100^ Managing expectations was deemed important as high expectations from targeted therapy led to difficulties in accepting eventual disease progression and end-of-life transitions^33,81^ Patients reported that testing can be empowering and overwhelming at the same time and also felt the need for developing centralized patient support systems^61^		Digital educational intervention improved patients and clinicians' sense of empowerment^26^ Online educational intervention might reduce cancer-related distress among patients^40^
Behavioral regulation (anything aimed at managing or changing objectively observed or measured actions)	Need for uniformity, accountability, and quality assurance of procedures and reporting^78^	Patients with cancer reported developing a unique identity associated with the cancer diagnosis that affects their information-seeking behavior and advocacy^81^		

#### Factors From the Clinicians' Perspective

Data were available for all 14 domains of the TDF for associated factors from clinicians' perspectives. The most commonly occurring and salient domains included knowledge, skills, social/professional roles, and environmental context and resources. Included studies reported limited and inconsistent knowledge and skills around biomarkers among health professionals who were not specialized in oncology.^41,49,51,53,59,66,69,70,72,80^ Among oncologists, knowledge was reported to be limited around how to order biomarker testing, interpreting test results, safety and efficacy profiles of immuno-oncology agents, making treatment recommendations based on the results of biomarker testing, and information sources to guide patients.^53,59,65^ Clinicians reported low self-efficacy in deciding whether biomarker testing was indicated, interpreting and communicating test results to the patients, explaining findings of uncertainty, and making treatment recommendations based on results.^43,51,53,57,65,74,92^ Clinicians reported uncertainties and low confidence in their patients' knowledge and ability to comprehend the biomarker test and its implications.^16,27,37^ Clinicians also reported limited skills in assessing patients' knowledge and information preferences, counseling and educating patients about implications, setting patient expectations, and communicating the rationale for treatment selection.^41,49,54,59,89^ With regard to their professional role and identity, clinicians reported that they were expected to be the gatekeepers for providing (or not providing) information and hence expected to inform the patients about treatment options based on the results of biomarker testing.^16,36,41,74^ Clinicians reported that the lack of expert consensus, professional recommendations, clinical guidelines, and protocols on the use of biomarker testing also contributed to uncertainties.^71-73,80^

Environmental contexts and resources also posed significant challenges, including excessive turnaround times for the results of biomarker testing,^16,29,32,64,97^ administrative requirements,^27,36^ lack of funding,^49,65^ lack of adequate staff,^66,84^ and high costs associated with infrastructure and technology.^16,37,69,70,73,84,88^ Clinicians also noted that lack of insurance coverage^16,31,54,65,66,69,79,84,97^ and issues with privacy or confidentiality^57,72,91^ may lead to patients being discriminated based on genomic information, and there were currently no ethical, legal, and social frameworks to manage these issues.^69^ Clinicians reported that limited time in clinics made it difficult for them to navigate clinical trials on biomarker testing.^41,54,59,92^ Concerns regarding social media were also highlighted, and clinicians reported that it sometimes led ineligible patients to feel that they were unfairly being denied access to an important test due to aggressive marketing over social media.^36,37,54,72,81^ Clinicians also raised concerns around patients' misperceptions of the likelihood of a cure or treatment side effects and noted that initial framing by clinicians sometimes sets high expectations for the benefits of targeted therapy for the patients.^54,81^ Clinicians acknowledged that patients' poor health could lead to a compromised understanding when burdened with stressful information and the details of the test results could result in information overload for some patients.^59,81,84,89^

Despite the uncertainties, clinicians were mostly optimistic and reported that biomarker testing was beneficial and worthwhile for the management of patients.^54,69,70,72,74,93^ Clinicians were intent on testing for biomarkers on the basis of clinical utility, that is, if it was likely to add value to the clinical decision-making process and was part of treatment guidelines, and if they had access to appropriate training and resources.^16,37,45,47,69-72,83^ Clinicians reported concerns around over-reliance on the results of biomarker testing,^37^ ordering inappropriately,^93^ uncertainty in validity,^54,72^ lack of evidence of benefit,^49,63,73,80^ or lack of evidence on the safety and efficacy of immuno-oncology agents.^59,95^ Some studies reported clinicians' reservations around testing for biomarkers when precision treatments were either not available or they did not have access to corresponding clinical trials, and hence, the results did not affect patients' treatment options.^49,54,73,78,89^

#### Factors From the Patients' Perspective

Data were available for all 14 domains of the TDF for factors associated with biomarker testing from the patients' perspective. The most commonly occurring and salient domains included knowledge, optimism, intentions, memory, attention and decision processes, environmental context and resources, social influences, and emotions. Patients reported lack of knowledge around biomarkers, what the test meant, how it related to their treatment, and the research processes.^30,60,67,73,86,87^ Moreover, patients often felt incompetent explaining biomarker testing to their relatives and highlighted the importance of understanding their results to communicate with relatives and reduce their uncertainties.^30,33,41^ Patients also acknowledged that the results were cognitively complex to understand, and it was therefore difficult to make decisions and hence could create unnecessary confusion and worry.^35,41^ The current communication around biomarker testing was reported to be limited and cursory.^33,41^ Patients preferred straightforward communication, education, and counseling to make informed decisions, with written information and time to review this information before decision making from their doctors.^34,42,45,47,83^ Patients preferred shared clinical decision making and saw their oncologist as a key partner in their decision-making process.^33,42^ However, receiving insufficient or quick information or the use of complex language to communicate results led to increased uncertainty about results and treatment recommendations.^16,30,33,41,42,61^ Sources of information about biomarker testing varied among the patients, whereas mass media was reported as a catalyst for access to information.^16,36,81^

Patients reported high expectations of the biomarker testing results and considered it a backup when other treatment approaches failed. For example, oncologists' framing of detection of a targetable mutation was reported as sometimes setting high expectations for patients.^33,81,87^ This, in turn, raised concerns around emotional burden associated with nonactionable or uncertain test results and their prognosis in the absence of targetable treatment options.^30,34,35,42,50^ Managing expectations was deemed important as high expectations from targeted therapy led to difficulty in accepting eventual disease progression and end-of-life transitions.^33,81^ Nonetheless, knowing the test results was reportedly more important than not knowing regardless of the potential anxiety and relieved treatment uncertainty to some degree.^30,100^

Despite the lack of knowledge and uncertainties, the majority of patients reported that they considered the biomarker test useful and expressed interest in taking the test.^33,42,60^ Patients were optimistic about contributing to advancing research to generate more reliable information that could be used to inform prognosis and improve survival even if efforts were unlikely to provide them with personal benefit.^30,33,42,67,73,85,90^ Patients expressed that the biomarker testing offered them more control, which was crucial as they already felt that they were not in control of their lives.^35,67^ Patients were willing to undergo the biomarker test given that it was covered by health insurance; however, they stated privacy concerns.^30,34,41,50,73,91,99^ Patients reported concerns around discrimination based on test results and hence preferred limited access to this information for their employers and insurance companies, whereas they were happy to share information with family members.^50,67^

#### Strategies to Facilitate Implementation

Setting up an institutional tumor board and multidisciplinary team coordination were the most frequently reported strategies to aid treatment decision making with clear lines of communication and better collaboration to facilitate implementation.^49,64,74,84,89,96^ Formal and ongoing education for clinicians^53,64,65,89^ and resources to increase genetic literacy among patients^17^ were suggested to increase knowledge, whereas online resources were put forward as a practical alternative to formal training sessions for the clinicians.^71^ Having institutional guidelines,^49^ setting up international academic exchange programs to train health professionals,^69^ trainings for laboratory technicians,^69^ educating physicians to provide better explanations to patients and communicating uncertainties,^54,71,89^ and electronic medical record alerts for genomic tests^98^ were suggested for improving skills. To aid shared clinical decision making, easy-to-understand patient education resources including pamphlets, websites, and scripts to communicate information related to biomarker testing and test results need to be developed.^59,83^ Advocacy and mobilization of funds and institutionalizing legal frameworks (protocols, policy and procedure manuals, and guidelines) were also suggested strategies to facilitate implementation.^69,89^

#### Effectiveness of Interventions to Facilitate Implementation

Seven studies evaluated interventions to facilitate implementation of biomarker testing: four studies evaluated educational interventions^26,28,40,55^; one study compared communicating results of biomarker testing with patients over phone versus in-person communication^39^; one study compared traditional static biomarker testing reports with interactive reports^52^; and one study evaluated the effectiveness of a virtual molecular and precision medicine clinic to improve access to genomic testing and clinical trials.^94^ Educational interventions were reported to be feasible, acceptable, and improved knowledge about precision oncology, clinical trials, and decisional self‐efficacy for targeted/immune therapy.^26,40,55^ One study reported that online accredited modules for clinicians enhanced knowledge, confidence, and intent to change practice.^28^ A web-based education program reported increased patient knowledge; however, there were no significant differences in distress outcomes.^40^ Using a virtual molecular and precision medicine clinic to improve access to genomic testing and clinical trials was reported to be an efficient means of offering real-time interpretation of genomic testing and identification of clinical trials for patients. It led to effective clinical trial enrollment and high rates of satisfaction among referring providers.^94^ Traditional static biomarker testing reports were found to be comparable with the interactive reports in terms of clinicians' overall comprehension scores. However, overall satisfaction scores were higher among clinicians in the interactive report group and clinicians exposed to the interactive report were more likely to correctly assess sequencing quality and understand when reports needed to be interpreted with caution.^52^ Disclosure of test results by telephone was found to be comparable with in-person communication.^39^

## DISCUSSION

This review summarizes findings from 77 studies across 14 domains of the TDF on factors associated with implementation of biomarker testing and strategies and interventions to facilitate implementation and improve clinical uptake. Overall, data on effectiveness of strategies and interventions to facilitate implementation and increase uptake of biomarker testing were scarce. Assessing the effectiveness of implementation strategies is challenging because of a myriad of reasons ranging from lack of validated biomarkers to its standardized integration into existing clinical practices. Lack of evidence on the effectiveness of implementation strategies reflects implementation challenges associated with awareness, communication, access, timely reporting, and funding.^101^

The challenges associated with integrating biomarker testing into clinical care have been documented previously whereby operational inefficiencies, limited understanding, inappropriate testing result usage, and access barriers lead to many patients not receiving the most effective personalized treatments in lung and colorectal cancers.^4,8^ Moreover, disparities in access owing to older age, treatment in community settings, low socioeconomic status, lower educational level, and area of residence have also been highlighted in the literature.^3^ Multiple factors contribute to the challenges of implementation and gaps in clinical practice given the complexity associated with biomarker testing. A review assessing evidence on health care professionals' attitudes toward precision oncology reported that despite the positive attitudes toward cancer precision medicine, there were concerns regarding costs, insurance coverage, limited knowledge about precision medicine, potential misuse, difficulties in accessing biomarker tests, and delays in receiving test results.^102^ Another review evaluating general public's knowledge, attitude, and motivation reported high awareness with little familiarity or factual knowledge with an overall positive attitude toward genomics.^103^ A scoping review of evidence on communication between patients and health care professionals regarding comprehensive biomarker testing reported that the patients had limited knowledge and understanding related to biomarker testing.^10^ Our review findings not only collate the existing evidence on factors associated with implementation but also highlight strategies and interventions with the potential to facilitate implementation, reduce implementation barriers, and improve clinical uptake. Findings from this review have implications for practice and future research. Institutional tumor boards play an increasingly crucial role in interpretation of the results of biomarker testing and consequently optimizing appropriate treatment recommendations.^104^ Future efforts need to be directed toward not only setting up institutional tumor boards but also standardizing their clinical integration through quality control measures. With the evolving landscape of biomarker validation and data repositories, digital platforms for data interpretation have the potential to improve understanding and implementation. Simultaneously, setting up institutional infrastructures to support multidisciplinary coordination also holds unparalleled significance. Any future research should happen in conjunction with building individual skills and capacity via ongoing education and dissemination to highlight the clinical utility of biomarker testing and availability of treatment options. Patient counseling related to biomarker testing and communicating test results, especially results of uncertainty, should be an integral component of skills development. There is a need to explore tools to assist clinicians in assessing patients' health literacy, facilitating conversations with patients, and understanding how best to communicate the test results that takes into consideration patients' anxieties and fears. Genetic counsellors might play a role in partnering with nongenetically trained clinicians to educate and facilitate communication about biomarker testing and test results.^105^ Finally, efforts should be made to evaluate the effectiveness of implementation strategies to mitigate barriers and increase access. Disparities in access and financial constraints remain major underlying issues hindering the clinical uptake of biomarker testing. Increasing insurance coverage could potentially improve the uptake; however, mitigation of confounding variables, specifically the complex interplay between race, ethnicity, and insurance, needs to be explored further.^4^ Interventions at the health systems level could potentially facilitate access especially in remote areas that lack tertiary care testing and treatment options. Considerations for future work may include identifying best practice models to establish linkages to connect individuals with health services and the existing health services with centers of excellence in health care in these areas and exploring opportunities to create infrastructure to integrate biomarker testing in remote and rural health care systems.^4,106^

This analysis has some strengths and limitations to consider when interpreting the findings. We conducted an all-inclusive search and independently assessed eligibility for full-text inclusion to decrease bias in the review process. Nevertheless, the possibility remains that we might have missed some studies. Included studies varied significantly in their design and quality and therefore can influence the overall findings as we did not exclude any study for quality reasons. Two reviewers, experienced in using TDF, ensured consistent interpretation of the domains when assigning the data extracted for factors associated with the implementation. However, there remains a possibility of potential over-reliance on predefined domains and the risk of overlooking other emergent themes. Complex interplay between various factors including demographics, socioeconomic status, level of educational, and cancer type, stage, or rarity may affect views, opinions, and preferences regarding biomarker testing which remains unexplored in this review. Consequently, we might not have fully captured the complexity of implementation barriers and facilitators associated with biomarker testing in diverse demographic and socioeconomic settings. Nonetheless, the findings from this review provide systematic up-to-date collated evidence on the factors associated with implementation of biomarker testing in cancer from both the provider and consumer perspective. It also synthesizes evidence-based strategies and interventions to facilitate implementation and improve clinical uptake and can assist in designing targeted strategies to reduce selected barriers to implementation and consequently increase access.

## APPENDIX 1. SEARCH STRATEGY

(((knowledge[MeSH Terms]) OR (comprehension[MeSH Terms]) OR (communication[MeSH Terms]) OR (“clinical decision making”[MeSH Terms]) OR (decision making, shared[MeSH Terms]) OR (implementation science[MeSH Terms]) OR (health plan implementation[MeSH Terms]) OR (“community health services”[MeSH Terms]) OR (clinical decision support systems[MeSH Terms]) OR (delivery of health care[MeSH Terms]) OR (practice guideline[MeSH Terms])) AND ((models, biologic[MeSH Terms]) OR (biological factors[MeSH Terms]) OR (biomarkers[MeSH Terms]) OR (“biomarkers, tumor”[MeSH Terms]) OR (“biomarkers, pharmacological”[MeSH Terms]) OR (“genetic markers”[MeSH Terms]) OR (genomics[MeSH Terms]) OR (“immunotherapy”[MeSH Terms]) OR (“precision medicine”[MeSH Terms])) AND (neoplasms[MeSH Terms]))

**TABLE A1.** Filters: Humans

Biomarkers		Knowledge, Understanding, Communication, and Implementation		Cancer
((models, biologic[MeSH Terms]) OR (biological factors[MeSH Terms]) OR (biomarkers[MeSH Terms]) OR (“biomarkers, tumor”[MeSH Terms]) OR (“biomarkers, pharmacological”[MeSH Terms]) OR (“genetic markers”[MeSH Terms]) OR (genomics[MeSH Terms]) OR (“immunotherapy”[MeSH Terms]) OR (“precision medicine”[MeSH Terms]))	AND	((knowledge[MeSH Terms]) OR (comprehension[MeSH Terms]) OR (communication[MeSH Terms]) OR (“clinical decision making”[MeSH Terms]) OR (decision making, shared[MeSH Terms]) OR (implementation science[MeSH Terms]) OR (health plan implementation[MeSH Terms]) OR (“community health services”[MeSH Terms]) OR (clinical decision support systems[MeSH Terms]) OR (delivery of health care[MeSH Terms]) OR (practice guideline[MeSH Terms]))	AND	(neoplasms[MeSH Terms])

**TABLE A2.** The Theoretical Domains Framework With Definitions and Component Constructs^18^

Domain	Constructs	Definitions
1. Knowledge	Knowledge (including knowledge of condition/scientific rationale) Procedural knowledge Knowledge of task environment	An awareness of the existence of something
2. Skills	Skills development Competence Ability Interpersonal skills Practice Skills assessment	An ability or proficiency acquired through practice
3. Social/professional role and identity	Professional identity Professional role Social identity Identity Professional boundaries Professional confidence Group identity Leadership Organizational commitment	A coherent set of behaviors and displayed personal qualities of an individual in a social or work setting
4. Beliefs about capabilities	Self-confidence Perceived competence Self-efficacy Perceived behavioral control Beliefs Self-esteem Empowerment Professional confidence	Acceptance of the truth, reality or validity about an ability, talent or facility that a person can put to constructive use
5. Optimism	Optimism Pessimism Unrealistic optimism Identity	The confidence that things will happen for the best or that desired goals will be attained
6. Beliefs about consequences	Beliefs Outcome expectancies Characteristics of outcome Expectancies Anticipated regret Consequents	Acceptance of the truth, reality, or validity about outcomes of a behavior in a given situation
7. Reinforcement	Rewards (proximal/distal, valued/not valued, probable/improbable) Incentives Punishment Consequents Reinforcement Contingencies Sanctions	Increasing the probability of a response by arranging a dependent relationship, or contingency, between the response and a given stimulus
8. Intentions	Stability of intentions Stages of change model Transtheoretical model and stages of change	A conscious decision to perform a behavior or a resolve to act in a certain way
9. Goals	Goals (distal/proximal) Goal priority Goal/target setting Goals (autonomous/controlled) Action planning Implementation intention	Mental representations of outcomes or end states that an individual wants to achieve
10. Memory, attention, and decision processes	Memory Attention Attention control Decision making Cognitive overload/tiredness	The ability to retain information, focus selectively on aspects of the environment, and choose between two or more alternatives
11. Environmental context and resources	Environmental stressors Resources/material resources Organizational culture/climate Salient events/critical incidents Person × environment interaction Barriers and facilitators	Any circumstance of a person's situation or environment that discourages or encourages the development of skills and abilities, independence, social competence, and adaptive behavior
12. Social influences	Social pressure Social norms Group conformity Social comparisons Group norms Social support Power Intergroup conflict Alienation Group identity Modeling	Those interpersonal processes that can cause individuals to change their thoughts, feelings, or behaviors
13. Emotion	Fear Anxiety Affect Stress Depression Positive/negative affect Burnout	A complex reaction pattern, involving experiential, behavioral, and physiological elements, by which the individual attempts to deal with a personally significant matter or event
14. Behavioral regulation	Self-monitoring Breaking habit Action planning	Anything aimed at managing or changing objectively observed or measured actions
